# Obesity Contributes to Inflammation in Patients with IBS via Complement Component 3 and C-Reactive Protein

**DOI:** 10.3390/nu14245227

**Published:** 2022-12-08

**Authors:** Pablo Thomas-Dupont, Henry Velázquez-Soto, Irma Yadira Izaguirre-Hernández, Mercedes Amieva-Balmori, Arturo Triana-Romero, Lorenzo Islas-Vázquez, María del Carmen Jiménez-Martínez, José María Remes-Troche

**Affiliations:** 1 Instituto de Investigaciones Médico-Biológicas, Universidad Veracruzana, Veracruz 91700, Mexico; 2 Departamento de Inmunología y Unidad de Investigación, Instituto de Oftalmología “Conde de Valencia”, Ciudad de México 06800, Mexico; 3 Departamento de Bioquímica, Facultad de Medicina, Universidad Nacional Autónoma de México, Ciudad de México 04510, Mexico

**Keywords:** irritable bowel syndrome, complement component 3, obesity

## Abstract

Irritable Bowel Syndrome (IBS) is usually a lifelong state that disturbs the digestive system. IBS has been linked to low-grade inflammation and the release of inflammatory mediators into the bloodstream. This could be associated with the degree of obesity presented by patients with IBS. Reports imply that IBS is more frequent in obese patients than in the overall population, with a prevalence of up to 31%. Here, we evaluated the serum levels of immunological and inflammation molecules and their correlation with Body Mass Index in IBS patients and the healthy control (HC). Seventy-nine serum samples of the IBS patients and thirty-five of the HC group were analyzed to determine the levels of each molecule and compare them with their BMI. Serum levels of C3 and C4 were significantly increased in IBS patients. C3 and C4 levels were higher in IBS-M and IBS-D subtypes compared with the HC group. When patients were grouped by BMI, a positive correlation between serum C3 (r = 0.49, *p* < 0.0001) and CRP (r = 0.40, *p* < 0.001) levels was found. Our results show, for the first time, a correlation between immunological molecules and BMI in IBS patients, suggesting that the inflammatory nature of obesity could contribute to the development of the symptoms in IBS through the stimulation and release of proteins as complement components and CRP.

## 1. Introduction

Irritable bowel syndrome (IBS) is a prevalent gastrointestinal (GI) disorder that affects between 5 and 18% of the adult population worldwide [1,2]. The condition is characterized by recurrent abdominal pain, mainly associated with alterations in the form or frequency of the stools [3]. IBS is the most frequent cause of gastroenterology consultations, and it has been well established that these patients display poor quality of life and are recurring healthcare system users [4]. The etiology of IBS is not completely elucidated, and several aspects have been implicated in the pathogenesis, such as altered gastrointestinal motility, intestinal inflammation, visceral hypersensitivity, post-infectious reactivity, disturbances in brain-gut interactions, modification in fecal microbiota, bacterial overgrowth, food sensitivity and malabsorption syndrome [5]. However, attention has focused on the role of low-grade mucosal inflammation due to the evidence that some patients with IBS have an increased number of inflammatory cells in the mucosa area [6]. The activity of GI nerves is modulated by the soluble mediators released by immune cells, causing symptoms in patients [7]. Obesity is another important factor that scientists have considered in the development of the disease. The prevalence rates of IBS range from 8 to 31% in obese patients, higher in these patients than in the general population [8,9,10]. The association between obesity and IBs has not been completely clarified. Obesity is a state of chronic low-grade inflammation that contributes to the development of dyslipidemia, insulin resistance, and hyperglycemia [11]. In addition, obesity is considered a risk factor for many functional GI disorders, including abdominal pain, bloating, heartburn, gastroesophageal reflux disease (GERD) symptoms and IBS [12], and body mass index (BMI) has been positively associated with abdominal pain and diarrhea [13]. The current study aimed to explore the serum levels of seven immunological and inflammation molecules and their correlation with BMI in IBS patients and healthy control (HC) subjects.

## 2. Materials and Methods

### 2.1. Population

We enlisted 79 Mexican patients that requested medical attention for gastrointestinal symptoms suggestive of IBS (abdominal pain and/or bloating) at the Instituto de Investigaciones Medico-Biológicas of the Universidad Veracruzana (a tertiary care center) who were prospectively evaluated from January to October 2019. Diagnosis of IBS was made according to the revised scoring system Rome IV for IBS (validated Spanish version). Patients who had an alarm sign (anemia, weight loss, fever, etc.) or a previous diagnosis of cancer, metabolic diseases such as diabetes mellitus, a history of major abdominal surgery (with the exception of cholecystectomy or appendectomy), thyroid disease, inflammatory bowel disease, celiac disease were excluded in the clinical evaluation. Patients with IBS were divided into three subgroups based on stool form: 24 (30.38%) were classified as IBS-diarrhea predominant (IBS-D), 29 (36.71%) as IBS-constipation predominant (IBS-C), and 26 (32.91%) as IBS-mixed pattern (IBS-M) [Table 1]. For the HC subjects, we included 35 serum samples from individuals of the general population. These subjects belonged to the same geographical region as the IBS patients. At the time of the sample collection, they did not exhibit any signs of illness, in accordance with physical examination and clinical evaluation. Additionally, HC subjects answered the Rome IV questionnaire to confirm the absence of IBS symptoms. Their samples were previously collected for population studies in the Veracruz-Boca del Río region at Centro Estatal de la Transfusión Sanguínea of Veracruz. We only included Mexican mestizos, defined as individuals born in Mexico and were the offspring of the native inhabitants of the region and individuals of Caucasian or Black African origin that arrived in America in the sixteenth century [14]. After a full explanation of the study, we obtained written informed consent from each participant in accordance with the guidelines of the Ethics Committee of the Instituto de Investigaciones Medico-Biológicas of the Universidad Veracruzana. The institutional ethics review board approved the study protocol number IIMB-UV 2019-12.

### 2.2. Measurement of Body Mass Index

BMI was calculated as the individual’s body weight (kg) divided by the square of their height (m). BMI was categorized according to the WHO classification of physical status [15]: underweight (BMI < 18.5 kg/m^2^), normal weight (BMI 18.5–25 kg/m^2^), overweight (25–30 kg/m^2^) or obese body condition (BMI > 30 kg/m^2^).

### 2.3. Serum Collection and Storage

Blood samples from patients with IBS or HC subjects were collected in a sterile vacuum tube without additives. The tubes were allowed to clot, then centrifuged at 3000× *g* rpm for 5 min to obtain or serum 500 μL aliquots were labeled and frozen at −80 °C for later use.

### 2.4. Serum Immunological Determinations

Serum Complement component 3 (C3), C4, Rheumatoid Factor (RF), C-Reactive Protein (CRP), Total Immunoglobulin (Ig)G, IgM, and IgA were determined using an automated chemistry analyzer COBAS C311 (Roche, Basilea, Switzerland).

### 2.5. Statistical Analysis

Data distribution normality was examined by the Kolmogorov–Smirnov test. The data were represented as medians with 25% and 75% interquartile ranges according to the data distribution. Non-parametric Mann–Whitney U test was used to compare two independent groups. Kruskal–Wallis (KW) test was used to compare three or more independent groups. When the KW test indicated a statistically significant difference, Dunn’s multiple comparison test was conducted to determine the sustained differences between the groups. Pearson’s correlation test was used to evaluate the correlation. GraphPad Prism v8.0.1 software was used to analyze the data. Differences between IBS and HC demographic data were calculated using the chi-squared test or unpaired *t*-test, as appropriate. *p* values < 0.05 were considered statistically significant.

## 3. Results

Table 1 summarizes the clinical and demographic data of the 79 patients diagnosed with IBS and the 35 healthy control subjects included in this study. All the subjects were categorized according to the BMI of WHO classification: underweight, normal, overweight, and obese.

### 3.1. Higher Serum Levels of C3 and C4 in IBS Patients Than in HC Group

**Figure 1:**
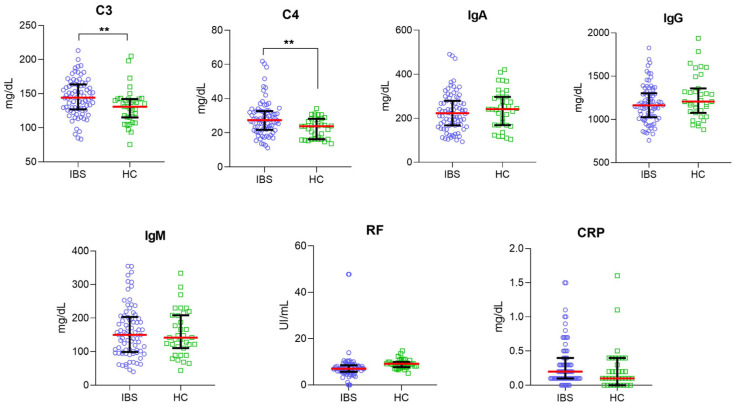
Comparison of the serum levels of C3, C4, IgA, IgG, IgM, RF, and CRP in IBS patients (*n* = 79; blue plots) and HC group (*n* = 35; green squared). A red-horizontal bar shows the median serum levels for each inflammatory marker. Error bars represent 25% and 95% percentile. Statistical significance was analyzed by Mann–Whitney U-test. The serum levels of each inflammatory marker are represented in mg/dL or UI/mL as appropriate; ** *p* = 0.0049 and *p* = 0.0023, respectively.

First, we evaluated seven serological markers of inflammation, such as C3 and C4, IgA, IgG, IgM, FR, and CRP in serum samples of IBS patients and HC subjects. Serum levels of C3 were significantly higher in IBS patients (median, [144 mg/dL]; range, [83.40 mg/dL–213.2 mg/dL]) than HC group (median, [131 mg/dL]; range, [75.60 mg/dL–205 mg/dL]; *p* < 0.001). Additionally, significant differences in the serum levels of C4 were found between IBS patients (median, [27.40 mg/dL]; range, [11.20 mg/dL–61.80 mg/dL]) and HC group (median, [23.80 mg/dL]; range, [13.70 mg/dL–34.20 mg/dL]; *p* < 0.001). There were no significant differences in IgA (IBS median, 223.3 mg/dL vs. HC median, 241.6 mg/dL; *p* = 0.3087), IgG (IBS median, 1165 mg/dL vs. HC median, 1206 mg/dL; *p* = 0.2106), IgM (IBS median, 150.2 mg/dL vs. HC median, 141.7 mg/dL; *p* = 0.8736), RF (IBS median, 7 UI/mL vs. HC median, 9.2 UI/mL; *p* = 0.0612) and CRP (IBS median, 0.2 mg/dL vs. HC median, 0.1 mg/dL; *p* = 0.1014) serum levels between IBS patients and HC group (Figure 1).

### 3.2. Higher Serum Levels of C3 and C4 in IBS Subgroups Than in HC Group

**Figure 2:**
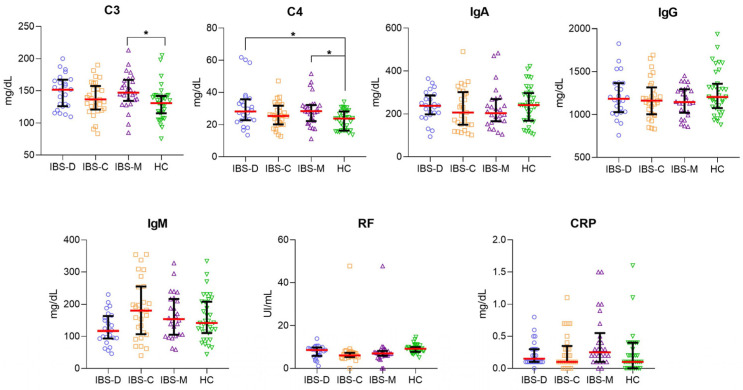
Comparison of the serum levels of C3, C4, IgA, IgG, IgM, RF and CRP in IBS patients classified according to stool form: IBS-D (*n* = 24, blue plots), IBS-C (*n* = 29; orange squared), IBS-M (*n* = 26; purple triangle) and HC (*n* = 35; green triangle). A red-horizontal bar shows the median serum levels for each inflammatory marker. Error bars represent 25% and 95% percentile. Kruskal–Wallis and Dunn’s multiple comparison tests were used to analyze the statistical significance. The serum levels of each inflammatory marker are represented in mg/dL or UI/mL as appropriate; * *p* < 0.05.

Given our previous results showing the higher serum levels of C3 and C4 in the IBS group, we classified IBS patients into three subgroups based on stool form: IBS-C, IBS-D, and IBS-M. In the IBS-M subgroup, the serum levels of C3 (median [147.2 mg/dL]; range [85.10 mg/dL–213.2 mg/dL]) and C4 (median [28.40 mg/dL]; range [11.20 mg/dL–51.60 mg/dL]) were significantly higher compared to the HC group (C3 median [131 mg/dL]; range [75.60 mg/dL–205 mg/dL]; *p* = 0.0224; C4 median [23.80 mg/dL]; range [13.70 mg/dL–34.20 mg/dL]; *p* = 0.0259). Additionally, C4 levels were significantly higher in the IBS-D subgroup (median [28.20 mg/dL]; range [13.50 mg/dL–61.8 mg/dL]) compared to the HC group (median [23.80 mg/dL]; range [13.70 mg/dL–34.2 mg/dL]; *p* = 0.0290). We did not find a statistically significant difference in the serum levels of IgA, IgG, IgM, RF, and CRP in the IBS subgroups and HC group (Figure 2).

### 3.3. Higher Serum Levels of C3 in IBS Women Than in HC Women

**Figure 3:**
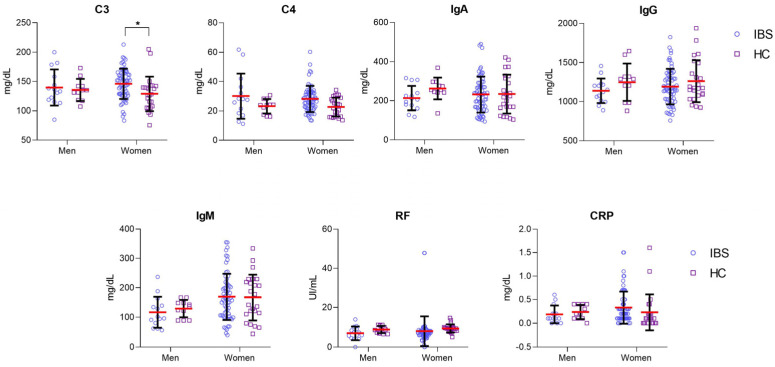
Higher serum levels of C3 in women with IBS (*n* = 65) than in the HC group (*n* = 24). Men groups (IBS, *n* = 14 and HC, *n* = 11). IBS groups were represented with blue plots, and HC groups with purple squared. A red-horizontal bar shows the median serum levels for each inflammatory marker. Error bars represent 25% and 95% percentile. Statistical significance was analyzed by Kruskal–Wallis and Dunn’s multiple comparison tests. The serum levels of each inflammatory marker are represented in mg/dL or UI/mL as appropriate; * *p* < 0.05.

It is well-established that immunological responses may be influenced by gender [16]. Next, we decided to evaluate the serum levels of all immunological markers according to the gender of patients with IBS and HC subjects. Serum levels of C3 were significantly higher in women with IBS (median, [147.8 mg/dL]; range, [83.40 mg/dL–213.2 mg/dL]) than in women of the HC group (median, [127.5 mg/dL]; range, [75.60 mg/dL–205 mg/dL]; *p* < 0.05). There were no significant differences in the rest of the markers (Figure 3).

### 3.4. Correlation Analysis between C3, C4, IgA, IgG, IgM, RF, and CRP Serum Levels and Body Mass Index in IBS and HC Groups

**Figure 4:**
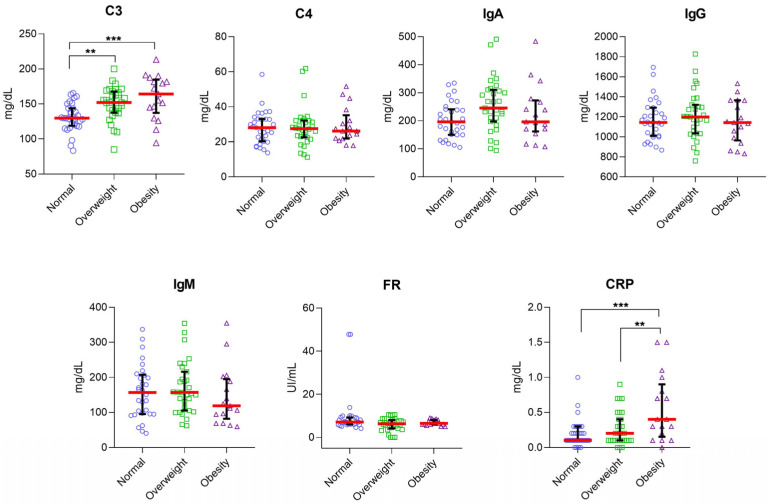
Serum levels of inflammatory markers in IBS patients according to BMI classification. The serum levels of C3and CRP were higher in the obesity group (*n* = 17; purple triangle) than in the overweight group (*n* = 31; green squared) and in the normal weight group (*n* = 31; blue plots). A red-horizontal bar shows the median serum levels for each inflammatory marker. Error bars represent 25% and 95% percentile. Statistical significance was analyzed by Kruskal–Wallis and Dunn’s multiple comparison tests. The serum levels of each inflammatory marker are represented in mg/dL or UI/mL as appropriate; ** *p* = 0.0042; *** *p* = 0.0010 in c3 comparisons, and ** *p* = 0.0046; *** *p* = 0.0004 in CRP comparisons.

**Figure 5:**
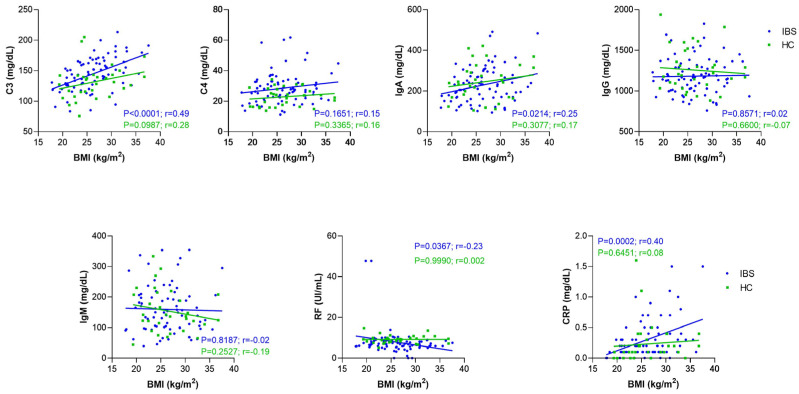
Correlation analysis between all serological inflammatory markers and BMI in patients with IBS (*n* = 79; blue plots) and HC group (*n* = 35; green plots). The lines represent the linear regression of each group. Correlations were carried out by Pearson’s correlation test. *p*-value < 0.05 was considered statistically significant.

**Figure 6:**
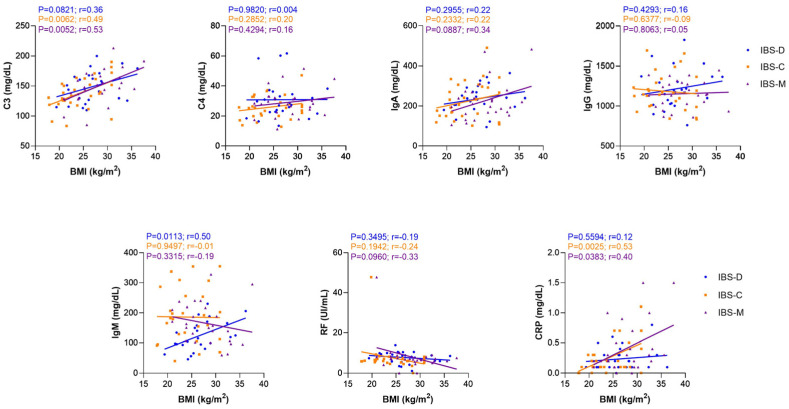
Correlation analysis between all serological inflammatory markers and BMI in IBS subgroups: IBS-D (*n* = 24; blue plots), IBS-C (*n* = 29; orange squared), and IBS-M (*n* = 26; purple triangle). The lines represent the linear regression of each group. Correlations were carried out by Pearson’s correlation test. *p*-value < 0.05 was considered statistically significant.

Although the relationship between chronic low-grade inflammation, insulin resistance, and other obesity-associated metabolic disorders is increasingly recognized, the mechanisms that activate these immunological processes are not entirely understood [17,18]. First, we categorized the IBS patients according to the BMI of WHO classification in underweight (0%), normal (39.24%), overweight (39.24%), and obesity (21.52%). Then, we evaluated the serum levels of the serological markers, and we found that serum levels of C3 were higher in patients with IBS classified as overweight (median [152.10 mg/dL]; range [85.10 mg/dL–200 mg/dL]) and obesity (median [164.30 mg/dL]; range [94.40 mg/dL–213.20 mg/dL]) than in IBS patients with normal weight (median [129.70 mg/dL]; range [83.40 mg/dL–165.40 mg/dL]; *p* = 0042 and *p* < 0.001). Differences in the serum levels of CRP were found between patients with overweight and obesity (*p* = 0.0046). In addition, the serum levels of CRP were statistically higher in patients with obesity versus patients with normal weight (*p* = 0.0004, Figure 4). Considering our previous results, we performed a correlation analysis between all the serological markers and BMI classification in both groups: IBS and HC. A significant positive correlation was found between C3 (r = 0.49, *p* < 0.0001), IgA (r = 0.25, *p* < 0.05), RF (r = −0.23, *p* < 0.05), and CRP (r = 0.40, *p* < 0.001) and BMI in the IBS group. No statistically significant correlation was found between the levels of C3 (r = 0.28, *p* = 0.0987), C4 (r = 0.16, *p* = 0.3365), IgA (r = 0.17, *p* = 3070), IgG (r = −0.07, *p* = 0.6600), IgM (r = −0.19, *p* = 0.2527), RF (r = 0.0002, *p* = 0.999), and CRP (r = 0.08, *p* = 0.6451) and BMI in the HC group (Figure 5). Additionally, we analyzed the correlation between serological markers and BMI in IBS subgroups (IBS-D, IBS-C, and IBS-M). We found that serum levels of C3 and CRP correlated with BMI in patients classified in IBS-C (C3, r = 0.49, *p* = 0.006; CRP, r = 0.53, *p* = 0.0025) and IBS-M (C3, r = 0.53, *p* = 0.0052; CRP, r = 0.40, *p* = 0.0383) subgroups (Figure 6).

## 4. Discussion

Obesity is associated with low-grade chronic inflammation that promotes chronic diseases, such as type 2 diabetes (T2D) [19], cardiovascular (CVD) [20], metabolic alterations [21], hypertension (HT) [22], nonalcoholic steatohepatitis (NASH) [23], and some types of cancer [24]. We evaluated the contribution of obesity to inflammation in IBS patients. Individuals that are overweight and obese have altered serum levels of inflammatory cytokines and other immune components [17]. It is well known that the complement system is essential in triggering an immune response. Moreover, it has extensive functions of promoting the clearance of apoptotic or necrotic cells, participating in tissue repair, and regulating inflammation. C3 is a soluble component that is a key element in all the complement activation pathways and the enhancement of inflammation. Meanwhile, complement component C4 turns into C3 convertase when it binds to C2b, which allows for C3 activation in the classical and mannan-binding within lectin pathways, two of the three activations of complement system pathways [25,26]. C3 is an acute-phase protein and can be involved in virtually any inflammatory condition.

Elevated levels of C3 and/or C3 cleavage products in the bloodstream were described in numerous pathologies, including autoimmune, neurological, renal, and cardiovascular diseases, cancer, transplant rejection, and obesity [27]. Uncontrolled and excessive complement activation may contribute to pathogenesis and enhance effects such as systemic inflammation, tissue damage, dysregulation of coagulation, and fibrinolysis, which emphasizes the importance of the tight regulation of complement activation in the maintenance of homeostasis. Regarding gastrointestinal inflammatory diseases, complement activation has been reported for chronic inflammatory bowel disease (IBD), such as Crohn’s disease (CD) or ulcerative colitis (UC). In this study, we provided information on the production of C3 and C4 in patients with IBS; to our knowledge, this is the first report that identified the production of these components of the complement system in this pathology. The liver is the main producer of the circulating complement components. Besides the liver, endothelial and epithelial cells are capable of complement production. In the intestine, complement factors have already been documented in the duodenum, occurring via the pancreatic compartment [28]. 

Furthermore, in the GI tract, intestinal epithelial cells (IECs) have also been identified as a source of distinct complement components [29]. In addition, diverse studies have shown the intestinal synthesis of complement receptors and complement-regulatory proteins. Further analyses are required to determine the sources of C3 and C4 complement components in IBS and their role in local inflammation. Several pieces of research provided information on a relationship between circulating complement components and metabolic disorders associated with obesity and demonstrated a possible role of complement system activation in adipose tissue physiology [30,31,32]. 

When we classify our study subjects according to their BMI as normal, overweight, or obese, levels of C3 increase as the BMI does. Although there are reports that establish that serum levels of complement C3 increase with obesity and correlate positively with BMI [33], this phenomenon is not observed in the controls but in the patients, suggesting, at least for our population, that the levels of circulating C3 are related to the IBS more than the obesity condition. Surprisingly, in the case of C4, conversely to previous studies [34], we did not find significant relationships between serum C4 levels and BMI in these patients. IBS could be differentiated from other causes of chronic diarrhea by the presence of pain that increases before defecation, alleviates after deposition, and is associated with changes in stool form or frequency [35]. Mechanistically, an imbalance in electrolyte absorption and/or secretion in the intestine disrupts the osmotic gradient, resulting in water retention in the lumen and diarrhea. This can be caused by a highly inflammatory milieu that can damage the mucosal architecture, compromising the epithelial barrier. Due to the inflammatory nature of C3 and C4, we classified the patients according to the stool form, IBS-D, IBS-C, and IBS-M and correlated to the BMI to identify if there is a different pattern production of these components of complement that can help to explain the most characteristic symptom in IBS. Interestingly, the production of C3 was higher in patients with IBS-M. The link between C3 and intestinal permeability has not been systematically or comprehensively studied, and the current understanding is incomplete. Zhang et al. mentioned that intracellular C3 deposition in intestinal epithelia contributes to the development of tissue damage [36]. C4 levels were elevated in IBS-D and IBS-M patients. Complement C4 is the most polymorphic protein in the complement system. Enough evidence demonstrates that individuals with C4 deficiency are susceptible to autoimmune disorders and microbial infections [37]. It is noteworthy that the IBS-C does not show significative differences in the production of these molecules, which could mean that they have a functional role in developing this characteristic. However, further investigation is required to demonstrate the participation of C3 and C4 in the permeability changes that give rise to the different phenotypes in IBS. We observed high levels of CRP in the serum of obese patients with IBS but not in the normal or overweight patients with IBS. CPR has long been a useful biomarker for systemic inflammations. CRP has been strongly associated with overweight and obesity in human epidemiological studies and is considered a consequence of obesity rather than the cause [38]. Previous reports have identified CRP in IBS patients with similar levels to HC subjects [39]; therefore, the increased level of this protein could not necessarily be specific to IBS but rather seems to be related to the BMI of the patient. CRP elevation can trigger a broad range of changes in the immune system, including activation of the complement system. In human serum, CRP binds to C1q and activates the classical pathway of the complement [40]. That could be a mechanism involved in the perpetuation of inflammation in IBS; however, many issues still need to be addressed. It is known that gender can influence and significantly impact the immune system [41]. In our IBS patients, levels of C3 were highest in women than in men, while C4 levels did not exhibit significant differences. It has previously been described that IBS has a predisposition related to sex or gender, presenting a higher prevalence in women with a ratio of 2–2.5:1 than in men, in terms of those who seek medical care [42,43]. Females and males have distinct innate and adaptive immune responses [44]. Generally, the immune response seems stronger in females than in males [41]. In addition to prevalence, various studies have described gender differences in the severity and duration of symptoms, differential production of soluble mediators such as cytokines, and response to treatment in IBS [45,46]. It has been published that levels of C3 are significantly lower in women compared to men, but those results were taken from healthy individuals with different backgrounds [47]. This behavior has been documented in other conditions [48]. However, the reasons why IBS is more prevalent in women than men and why women experience greater severity of symptoms, have an increased inflammatory cytokine, and have an impaired quality of life compared with men remain to be explored in detail. Regarding obesity as a factor that can explain the differences in C3 production according to sex, Karkhaneh et al. published that obese women with high body fat percentages have higher serum C3 than healthy, non-obese ones [49]. Finally, it is important to mention that we considered it relevant to measure proinflammatory markers such as C3, C4, RF, CRP, IgG, IgM, and IgA as a first screening since IBS presents an underlying immunological condition [50,51], but no molecule is a specific biomarker for IBS. Further investigations are necessary to understand the role of these molecules in the immunopathology of IBS.

## 5. Conclusions

From our perspective, the inflammatory nature of obesity could contribute to developing the symptoms of IBS through the stimulation and release of proteins as complement components and CRP. Nevertheless, this finding raises further questions about the mechanisms involved and their regulation.

## Figures and Tables

**Table 1:** Demographic characteristics of the participating subjects.

	IBS-Rome IV (*n* = 79)	Healthy Controls (*n* = 35)	*p* Value
Sex			
Women, *n* (%)	65 (82.27)	24 (68.57)	0.1403 †
Men, *n* (%)	14 (17.73)	11 (31.43)	
Age (mean, SD)	32.72 ± 13.28	35.24 ± 10.86	0.9151 *
Body mass index (mean, SD)	26.26 ± 4.5	26.10 ± 4.66	0.617 *
Length of time of symptom progression (mean in months, SD)	82.47 ± 91.64	—	—
Weight			
Underweight, *n* (%)	0	0	
Normal, *n* (%)	31 (39.24)	18 (51.42)	0.2313 †
Overweight, *n* (%)	31 (39.24)	8 (22.85)	
Obesity, *n* (%)	17 (21.52)	9 (25.71)	
IBS subtype			
IBS-C, *n* (%)	29 (36.71)	—	—
IBS-M, *n* (%)	26 (32.91)	—	—
IBS-D, *n* (%)	24 (30.38)	—	—

IBS, Irritable Bowel Syndrome, IBS-C, Irritable Bowel Syndrome-constipation predominant; IBS-M, Irritable Bowel Syndrome-mixed pattern; IBS-D, Irritable Bowel Syndrome-diarrhea predominant. † Chi-squared test. * Unpaired *t*-test.

## Data Availability

Not applicable.
